# Computational model of collective nest selection by ants with heterogeneous acceptance thresholds

**DOI:** 10.1098/rsos.140533

**Published:** 2015-06-09

**Authors:** Naoki Masuda, Thomas A. O'shea-Wheller, Carolina Doran, Nigel R. Franks

**Affiliations:** 1 Department of Engineering Mathematics, Merchant Venturers Building, University of Bristol, Woodland Road, Clifton, Bristol BS8 1UB, UK; 2 School of Biological Sciences, University of Bristol, Life Sciences Building, 24 Tyndall Avenue, Bristol, England, BS8 1TQ, UK; 3 Champalimaud Neuroscience Programme, Champalimaud Centre for the Unknown, Avenida Brasília, Lisbon 1400-038, Portugal

**Keywords:** collective decision-making, wisdom of crowds, social insects, response thresholds

## Abstract

Collective decision-making is a characteristic of societies ranging from ants to humans. The ant *Temnothorax albipennis* is known to use quorum sensing to collectively decide on a new home; emigration to a new nest site occurs when the number of ants favouring the new site becomes quorate. There are several possible mechanisms by which ant colonies can select the best nest site among alternatives based on a quorum mechanism. In this study, we use computational models to examine the implications of heterogeneous acceptance thresholds across individual ants in collective nest choice behaviour. We take a minimalist approach to develop a differential equation model and a corresponding non-spatial agent-based model. We show, consistent with existing empirical evidence, that heterogeneity in acceptance thresholds is a viable mechanism for efficient nest choice behaviour. In particular, we show that the proposed models show speed–accuracy trade-offs and speed–cohesion trade-offs when we vary the number of scouts or the quorum threshold.

## 1. Introduction

Consensus decision-making is common among various animal groups [1–3] and is particularly prevalent in eusocial species, in which group behaviour often confers abilities above and beyond those of individuals [4]. To make a group decision, individual animals often communicate to exchange information, possibly under the influence of external inputs or changing environments. Quorum sensing, whereby an individual begins an action only when the density of conspecifics exceeds a threshold, is a mechanism often used in consensus decision-making. It has long been known that quorum sensing is involved in gene expression in bacteria, serving to regulate physiological activities and extracellular virulence factors [5,6]. However, metazoans, including social insects and various vertebrates, also show quorum responses [7,3]. Examples include selection of nest sites by ants [8,9], honeybees [10] and bats [11], following behaviour of fish (sticklebacks) [12,13], decisions to leave a foraging site by meerkats [14], movement in a new direction by macaques [15] and even gaze following by human crowds [16,17]. Importantly, such a quorum response is useful for accurate decision-making because a certain number of individuals must separately deem an option to be suitable before the group can collectively become quorate for that option. Quorum sensing serves to pool information from multiple individuals in order to overcome errors inherent at the level of individual decision-making [1–3].

An example of consensus decision-making using quorum responses can be seen in the ant *Temnothorax albipennis* during nest choice behaviour, whereby when the number of ants favouring a new nest site exceeds a quorum threshold, the colony collectively chooses to emigrate to that site. Specifically, once this threshold is attained, colonies switch from relatively slow recruitment behaviour to rapid transport behaviour, allowing expedited migration to their nest of choice [8].

Previous experimental work suggests that, rather than comparing directly between nests, each ant may have a fixed internal quality threshold against which it assesses potential new homes, and that this threshold varies among individuals within a colony [18,19]. In fact, it has been found that when choosing a new nest site certain individual ants will continually reject a new nest site of lower quality, even if barred from making direct comparisons with other nest sites of higher quality, while other individuals in the same colony seem satisfied and will choose the lower quality nest site [20]. Such collective decision-making, based on heterogeneously distributed thresholds, may provide a robust and adaptable method of choosing between nests varying in quality [19]. Heterogeneity in the response thresholds of animals has also been observed in different scenarios. Honeybees [21] and bumblebees [22,23] in a single colony are heterogeneous in their response thresholds to environmental fluctuation. Honeybees are also heterogeneous in their proboscis extension response thresholds for sucrose [24], and bumblebees have heterogeneous responses to odours [25], with the latter being induced by heterogeneity in body size, especially in the size of their antennae. In addition, a famous computational model describing behavioural cascades for humans, which assumes heterogeneous quorum thresholds across individuals [26], also has empirical support in this respect [27].

An earlier computational study has used a spatially explicit agent-based model to demonstrate the viability of the heterogeneous acceptance threshold hypothesis in collective nest choice [19]. Crucially, it was shown that nest-dependent recruitment latency could be explained as a by-product of heterogeneous acceptance thresholds. The goal of the current study is to explore this mechanism further, by introducing non-spatial mathematical models taking a minimalist approach (i.e. with a relatively small number of variables and parameters). Using these models, we explore the efficiency of nest choice behaviour depending on the number of high-threshold ants, quorum threshold, the rate at which ants convert to recruiters and so on. In particular, we focus on the possibility of speed–accuracy trade-offs [9,28,29] and speed–cohesion trade-offs [30]. Speed–accuracy trade-offs represent those between the speed at which a collective decision is made and the probability with which a good nest site is selected among candidate sites. For example, under harsh conditions, ants lower quorum thresholds and may accept relatively low-quality nests in order to prioritize speed [9]. Speed–cohesion trade-offs represent those between the decision speed and the extent to which a group remains together as a single entity when candidate nest sites are equally good. For example, a colony sacrifices its unity in the emigration process when the home nest is destroyed [30]. Although emigration occurs rapidly in this case, accuracy is not compromised by the act of splitting because all nest sites were of the same quality, making accuracy an irrelevant issue. Instead, the unity of the colony is compromised.

## 2. Model

Our models concern collective nest choice between two new nest sites, one of which is good and the other poor. Experimentally, two (or more) nests of different qualities are presented to a colony, with the quality of each nest as the ants perceive it depending on floor area, ceiling height, darkness and entrance size [31]. Even if the current nest is kept intact, as we assume in the following models, colonies emigrate if a sufficiently better nest site is presented [32]. All ants initially stay in the current nest. The colony starts exploring the new nest sites by sending out some scouts (i.e. ants that proactively search for suitable sites) and later may recruit all of the remaining ants in the colony. The construction of the model consists of two stages. First, we define a deterministic model composed of a set of ordinary differential equations in which each variable represents a type of ant in a particular state. The variables are listed in table 1. The differential equation model corresponds to an infinite population in which two types of ant, low-threshold and high-threshold ones, are mixed. Second, we derive from the differential equation model an agent-based stochastic model. The latter model corresponds to a finite population and shows stochastic behaviour, which is relevant to speed–accuracy trade-offs. Otherwise, the two models are the same; they have the same number of variables and assume the same behavioural rules for ants. When we assume an infinite population in the agent-based model, the stochastic effect is averaged away such that the agent-based model is reduced to the differential equation model. Both models do not have spatial structure, and we neglect the travel time between nest sites.

**Table 1.** Variables for the differential equation model.

variable	meaning
*x*_ℓ,c_	low-threshold ants in the current nest
*x*_c_	ants in the current nest (=*x*_ℓ,c_+*x*_h,c_)
*x*_h,p,vis_	high-threshold ants temporarily visiting the poor nest site
*x*_ℓ,p,com_	low-threshold ants committed to the poor nest site
*x*_ℓ,g,com_	low-threshold ants committed to the good nest site
*x*_g,com_	ants committed to the good nest site (=*x*_ℓ,g,com_+*x*_h,g,com_)
*x*_ℓ,p,rec_	low-threshold ants recruiting to the poor nest site
*x*_ℓ,g,rec_	low-threshold ants recruiting to the good nest site
*x*_g,rec_	ants recruiting to the good nest site (=*x*_ℓ,g,rec_+*x*_h,g,rec_)

### 2.1 Differential equation model

Ants are assumed to have either of the two thresholds, i.e. low or high. The low threshold implies that an ant accepts both good and poor nest sites. The high threshold implies that an ant accepts only the good nest site. We denote the fraction of the ants with high and low thresholds by *H* and *L*, respectively. We have *H*+*L*=1. By changing the *H* value (equivalently, *L* value), we can control the mean threshold, or mean choosiness of the population.

**Figure 1.**
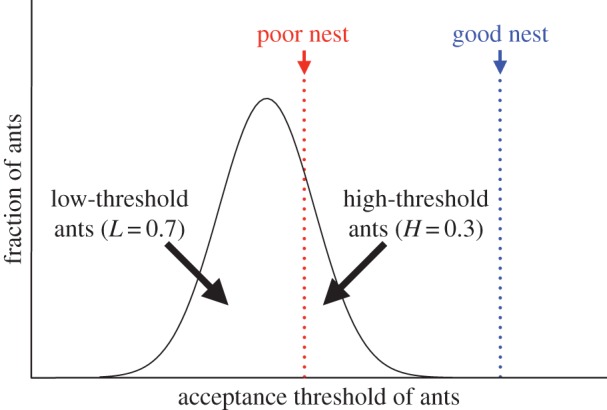
The normal distribution of the threshold can be mapped to a two-valued threshold distribution when there are two nest sites. The fraction of ants having a threshold larger and smaller than the quality of the poor nest can be identified with *H* and *L*, respectively.

In a previous model, we assumed that the threshold is distributed according to a normal distribution [19], which may seem more plausible than the two-point distribution employed in this study. However, when there are just two new nest sites of different quality, the results are not affected by the distribution of thresholds except in pathological cases. Figure 1 illustrates this point. If we assume that all ants are satisfied with the better nest, *H* in our model is identical to the fraction of ants having a threshold larger than the quality of the poor nest in the normal distribution model. The fraction of ants whose threshold is smaller than the quality of the poor nest corresponds to *L*.

Ants are in either the current nest, poor new site, or good new site. We denote by *x*_ℓ,c_ the fraction of ants that have a low threshold and are in the current nest. *x*_ℓ,p,rec_ is the fraction of ants that have a low threshold, are in the poor nest site and are recruiting. *x*_ℓ,p,com_ is the fraction of ants that have a low threshold, are in the poor site and are committed to (but not recruiting to) the poor site (table 1). Variables *x*_h,c_, *x*_ℓ,g,rec_, *x*_h,g,rec_, *x*_ℓ,g,com_ and *x*_h,g,com_ are similarly defined. In addition, we denote by *x*_h,p,vis_ the fraction of ants that have a high threshold and are visiting, but not committed to, the poor nest site. These ants will not commit to the poor nest site because they will never be satisfied with it.

The dynamics for ants in the current nest are given by

2.1 $$\frac{dx_{\ell ,c}}{dt}=-(x_{\ell ,p,\mathrm{rec}}+x_{\ell ,g,\mathrm{rec}}+x_{h,g,\mathrm{rec}})x_{\ell ,c}+\alpha_{\mathrm{leak}}(x_{\ell ,p,\mathrm{com}}+x_{\ell ,p,\mathrm{rec}}+x_{\ell ,g,\mathrm{com}}+x_{\ell ,g,\mathrm{rec}})$$

and

2.2 $$\frac{dx_{h,c}}{dt}=-(x_{\ell ,p,\mathrm{rec}}+x_{\ell ,g,\mathrm{rec}}+x_{h,g,\mathrm{rec}})x_{h,c}+\alpha_{\mathrm{leak}}(x_{h,p,\mathrm{vis}}+x_{h,g,\mathrm{com}}+x_{h,g,\mathrm{rec}}),$$

where *t* denotes the time. *x*_ℓ,c_ and *x*_h,c_ correspond to the ants searching for new sites assumed in the model proposed in Pratt *et al.* [8]. The first terms on the right-hand side of equations (2.1) and (2.2) represent the assumption that ants in the current nest are recruited to the good or poor nest sites by recruiters. In these terms, *x*_ℓ,p,rec_+*x*_ℓ,g,rec_+*x*_h,g,rec_ is equal to the total fraction of recruiters. They are recruiting the ants in the current nest to the poor or the good nest site. The frequency at which recruitment occurs is proportional to the fraction of recruiters and the fraction of recruitees, according to the usual mass interaction rule. In other words, each ant in the current nest is successfully recruited to either new nest site at a rate equal to the number of recruiters, normalized by the population size that we do not explicitly model here. The second terms on the right-hand side of equations (2.1) and (2.2) represent the effect of leakage; *α*_leak_ is the leakage rate. The leakage implies that ants committed or recruiting to a new nest site may stop doing so and return to the current nest. Such ants may later be recruited to a different nest and become committed to it. We introduce the leakage terms because previous studies assumed leakage in one form or another to allow for ants to switch from one new nest site to another and back [8,33–37].

Low-threshold ants commit to the poor nest site once recruited. Therefore, we assume

2.3 $$\frac{dx_{\ell ,p,\mathrm{com}}}{dt}=x_{\ell ,p,\mathrm{rec}}x_{\ell ,c}-\alpha_{p}x_{\ell ,p,\mathrm{com}}-\alpha_{\mathrm{leak}}x_{\ell ,p,\mathrm{com}}.$$

The first term on the right-hand side of equation (2.3) comes from the successful recruitment and corresponds to a quantity on the right-hand side of equation (2.1). The second term accounts for conversion from the committed state to recruiter. Parameter *α*_p_ represents the rate at which the committed individual turns into a recruiter. In other words, the committed individual waits for characteristic time 1/*α*_p_ before turning into a recruiter. The third term represents the leakage.

High-threshold ants recruited to the poor nest site are unsatisfied. Therefore, we assume that the fraction of high-threshold ants visiting, but not committed to, the poor nest site obeys the following dynamics:

2.4 $$\frac{dx_{h,p,\mathrm{vis}}}{dt}=x_{\ell ,p,\mathrm{rec}}x_{h,c}-\alpha_{s}x_{h,p,\mathrm{vis}}-\alpha_{\mathrm{leak}}x_{h,p,\mathrm{vis}}.$$

The rate at which an individual switches to the good nest site is represented by *α*_s_. In other words, high-threshold ants wait for characteristic time 1/*α*_s_ before switching to the good nest site.

All ants recruited to the good nest site and high-threshold ants that have switched from the poor to good nest site are satisfied and get committed to the good nest site. Therefore, we obtain

2.5 $$\frac{dx_{\ell ,g,\mathrm{com}}}{dt}=(x_{\ell ,g,\mathrm{rec}}+x_{h,g,\mathrm{rec}})x_{\ell ,c}-\alpha_{g}x_{\ell ,g,\mathrm{com}}-\alpha_{\mathrm{leak}}x_{\ell ,g,\mathrm{com}}$$

and

2.6 $$\frac{dx_{h,g,\mathrm{com}}}{dt}=(x_{\ell ,g,\mathrm{rec}}+x_{h,g,\mathrm{rec}})x_{h,c}+\alpha_{s}x_{h,p,\mathrm{vis}}-\alpha_{g}x_{h,g,\mathrm{com}}-\alpha_{\mathrm{leak}}x_{h,g,\mathrm{com}},$$

where *x*_ℓ,g,com_ is the fraction of ants that have a low threshold and are committed to the good nest site, and similarly for *x*_h,g,com_. Parameter *α*_g_ represents the rate at which an ant committed to the good nest site starts recruiting. This rate is assumed to be common to low- and high-threshold ants.

Finally, committed individuals turn into recruiters as follows:

2.7 $$\begin{array}{rl} \frac{dx_{\ell ,p,\mathrm{rec}}}{dt} & =\alpha_{p}x_{\ell ,p,\mathrm{com}}-\alpha_{\mathrm{leak}}x_{\ell ,p,\mathrm{rec}}, \end{array}$$

2.8 $$\begin{array}{rl} \frac{dx_{\ell ,g,\mathrm{rec}}}{dt} & =\alpha_{g}x_{\ell ,g,\mathrm{com}}-\alpha_{\mathrm{leak}}x_{\ell ,g,\mathrm{rec}}, \end{array}$$

2.9 $$\begin{array}{rl} \mathrm{and}\;\frac{dx_{h,g,\mathrm{rec}}}{dt} & =\alpha_{g}x_{h,g,\mathrm{com}}-\alpha_{\mathrm{leak}}x_{h,g,\mathrm{rec}}. \end{array}$$

Ants that have once been committed to either nest site always become recruiters for the corresponding nest site, except in the case that they become uncommitted to this nest due to leakage, and thus return to the current nest. Committed ants in our model are similar to assessors in the previous models [8,34,35]. The difference is that assessors in the previous models [8,34,35] are allowed to directly switch to other new sites, whereas committed ants in our model have to return to the current nest via leakage before switching to a different new site. In the present model, direct switching occurs only for high-threshold ants visiting the poor nest site. This class of ant cannot be identified with assessors, either, because they will never commit to the poor nest site.

**Figure 2.**
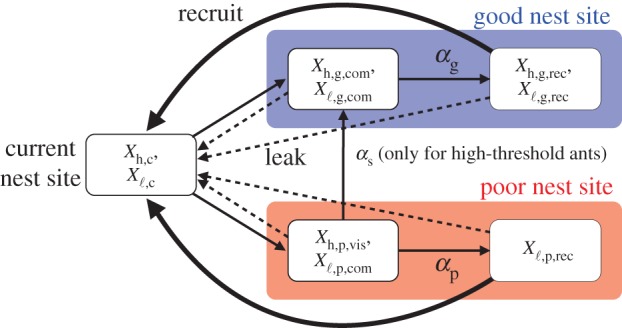
Schematic illustration of the model. The thin solid lines represent the state transitions except those induced by leakage. The thick solid lines represent the effect of recruitment exerted on ants in the current nest. The dotted lines represent the state transitions induced by leakage.

To summarize the model, it is a set of differential equations with nine variables. The model is schematically illustrated in figure 2. Because of the following two constraints:

2.10 $$x_{\ell ,c}+x_{\ell ,p,\mathrm{com}}+x_{\ell ,p,\mathrm{rec}}+x_{\ell ,g,\mathrm{com}}+x_{\ell ,g,\mathrm{rec}}=L$$

and

2.11 $$x_{h,c}+x_{h,p,\mathrm{vis}}+x_{h,g,\mathrm{com}}+x_{h,g,\mathrm{rec}}=H,$$

the model is seven-dimensional.

As the initial condition, we assume that a fraction *z* of ants are scouts, half going to the good nest site and the other half to the poor nest site. All other ants are initially located in the current nest. Therefore, we set *x*_ℓ,c_=*L*(1−*z*), *x*_ℓ,p,com_=*x*_ℓ,g,com_=*Lz*/2, *x*_h,c_=*H*(1−*z*), *x*_h,p,vis_=*x*_h,g,com_=*Hz*/2, and the other variables are equal to zero when *t*=0. Empirically, *z*≈0.3 or so [28].

We introduced two parameters *α*_p_ and *α*_g_; assuming *α*_g_>*α*_p_ corresponds to the recruitment latency hypothesis, according to which ants visiting a nest site hesitate for longer before recruiting nest-mates if the site is of low quality [38,34]. Although we do not explore the effect of heterogeneous recruitment latency in the following, our formulation allows it. If we set *H*=0 and hence assume that any ant is satisfied with either new nest site, we can examine the pure effect of the recruitment latency hypothesis without nest switching. Otherwise, we can examine the combined effect of the recruitment latency hypothesis and the heterogeneous acceptance threshold. In the following, we set *α*≡*α*_g_=*α*_p_ to focus on the threshold rule.

### 2.2 Agent-based model

In experiments, a colony might select the good nest site, select the poor site, or end up splitting between different sites. If there are two new nest sites of different quality, the accuracy of the nest choice is equivalent to the probability that the colony emigrates to the good nest site. The differential equation model presented in §2.1 cannot model such partly stochastic nest choice. In this section, we derive a finite-population variant of the differential equation model, which is agent-based and stochastic in nature. A similar conversion of a different-equation model into the corresponding stochastic agent-based model was employed in Shaffer *et al.* [37]. With our agent-based model, we explore probabilistic nest choice and the trade-offs between speed and accuracy.

We denote the number of ants by *N*. In the finite-population agent-based model, we interpret equation (2.1) as follows. Each low-threshold ant in the current nest will be recruited to the poor nest site by a low-threshold recruiter with rate *x*_ℓ,p,rec_=*N*_ℓ,p,rec_/*N*, where *N*_ℓ,p,rec_ is the number of low-threshold recruiters to the poor nest site. The rate refers to that of the Poisson process associated with this event. Equivalently, successful recruiting occurs with probability *x*_ℓ,p,rec_Δ*t*(=*N*_ℓ,p,rec_Δ*t*/*N*) for time period Δ*t*, when Δ*t* is small. Similarly, a low-threshold ant in the current nest is recruited to the good nest site by low-threshold recruiters with rate *x*_ℓ,g,rec_=*N*_ℓ,g,rec_/*N*, where *N*_ℓ,g,rec_ is the number of low-threshold recruiters to the good nest site. A low-threshold ant in the current nest is recruited to the good nest site by high-threshold recruiters with rate *x*_h,g,rec_=*N*_h,g,rec_/*N*, where *N*_h,g,rec_ is the number of high-threshold recruiters to the good nest site. Last, a low-threshold ant visiting either new nest returns to the current nest with rate *α*_leak_. In other words, this event occurs with probability *α*_leak_Δ*t* for time period Δ*t* for each ant. Equivalently, the time that a low-threshold ant spends before returning to the current nest obeys the exponential distribution with mean *α*^−1^_leak_, if the ant does not experience any other event in this time period. In the limit N→∞, the stochasticity is averaged away, and the dynamic of the low-threshold ants in the current nest subjected to the three types of recruitment is described by equation (2.1).

The other rules with which ants switch state are similarly redefined for the agent-based model. For example, based on the first term on the right-hand side of equation (2.7), we assume that a low-threshold ant committed to the poor nest site turns into a recruiter with rate *α*_p_(=*α*). In the limit N→∞, the entire agent-based model is the same as the differential equation model.

In accordance with the differential equation model, we give the initial condition by *N*_ℓ,c_=*NL*(1−*z*), *N*_ℓ,p,com_=*N*_ℓ,g,com_=*NLz*/2, *N*_ℓ,p,rec_=*N*_ℓ,g,rec_=0, *N*_h,c_=*NH*(1−*z*), *N*_h,p,vis_=*N*_h,g,com_=*Hz*/2, and *N*_h,g,rec_=0.

## 3. Results

### 3.1 Analytical results

**Figure 3.**
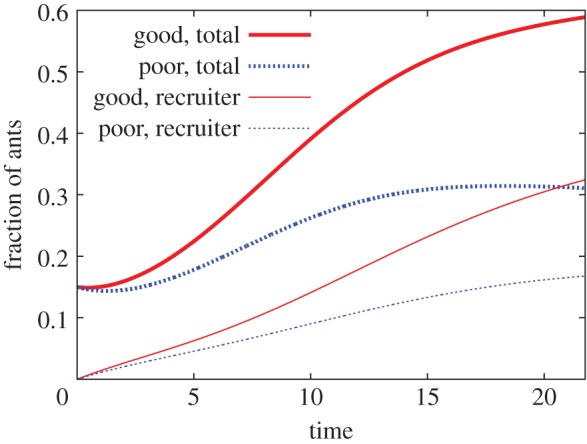
A sample time course of the differential equation model. We set *α*=0.1, *α*_s_=0.1, *α*_leak_=0.05, *H*=0.2 and *z*=0.3. The Euler scheme with time step 0.001 was used for numerical integration.

The fraction of the ‘vote’ (i.e. number of ants) for the good nest site and that for the poor nest site are given by *x*_ℓ,g,com_+*x*_h,g,com_+*x*_ℓ,g,rec_+*x*_h,g,rec_ and *x*_ℓ,p,com_+*x*_ℓ,p,rec_+*x*_h,p,vis_, respectively. In the differential equation model, the former is always larger than the latter for any *t* (see appendix A for the proof). Therefore, for an arbitrary quorum threshold, the quorum is reached in the good nest site before it is reached in the poor nest site. Note that the high-threshold ants temporarily visiting the poor nest site (without commitment) are also counted as votes for the poor site, consistent with experiments [18]. Even with this conservative counting of the vote, the good nest site is always chosen over the poor one. As corollaries, the numbers of committed ants and recruiters are always larger for the good nest site than for the poor nest site (appendix A). A sample time course of the differential equation model is shown in figure 3. Consistent with this theory, the vote for the good nest site and the corresponding number of recruiters are larger than those for the poor site.

### 3.2 Numerical results

We set *N*=100 and the quorum threshold to 0.5*N* unless otherwise stated. We set *α*_leak_=0.05 throughout the following numerical simulations. This leakage rate is fairly large in the cases in which the values of *α* or *α*_s_ are comparable to or smaller than the *α*_leak_ value. We also confirmed that the following results were qualitatively the same when we turned off the leakage, i.e. *α*_leak_=0 (electronic supplementary material, figure S1). We carried out 10^4^ runs of simulations for each set of parameter values and initial condition excluding runs in which the emigration was unsuccessful. Unsuccessful emigrations occurred when all ants happened to return to the current nest due to leakage such that the dynamic terminated. The following results are averages over the 10^4^ runs in which a new site has become quorate. We carefully selected the parameter values such that the initial number of ants in each category specified in §2.2 was integer and summed up to *N*. Then, we measured *T*, the time until the quorum threshold was reached for either new nest site, and *P*, the accuracy quantified by the fraction of runs in which the colony selected the good nest site.

**Figure 4.**
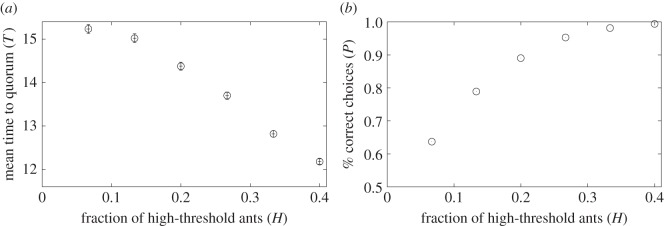
(*a*) Mean time to quorum, *T*, and (*b*) fraction of the correct choices, *P*, in the finite population model when we varied the fraction of high-threshold ants, *H*. We set *N*=100, *α*=0.1, *α*_s_=0.1, *α*_leak_=0.05 and *z*=0.3. The error bars in (*a*) represent the confidence intervals calculated as $1.96\times (\text{s.d.})/\sqrt{\text{number of runs}}$. The size of the error bars is at most approximately the same as that of the symbols showing the mean values. Error bars are not shown in (*b*) because we have used all runs to calculate a single p-value. The results shown in this and the following figures are calculated on the basis of 10^4^ runs.

The dependence of *T* and *P* on the fraction of high-threshold ants, *H*, is shown in figure 4 with *α*=0.1, *α*_s_=0.1 and *z*=0.3. We set *z* at 0.3 based on empirical findings [28]. Both the speed of the collective decision, which is proportional to 1/*T*, and the accuracy increased as *H* increased.

**Figure 5.**
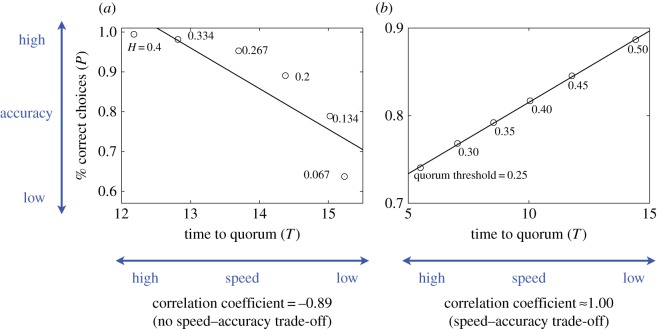
Calculation of the correlation coefficient. The relationship between the mean time to quorum, *T*, and the fraction of the correct choices, *P*, for the data in figure 4 is shown in (*a*). The linear regression applied to the six data points, corresponding to the six *H* values examined in figure 4, yields the line shown in the figure and the Pearson correlation coefficient of −0.89. In fact, the speed (i.e. 1/*T*) and accuracy (i.e. *P*) simultaneously increase or decrease as we vary *H*, which is consistent with the negative correlation coefficient value. A negative correlation coefficient value indicates an absence of speed–accuracy trade-offs. The relationship between *T* and *P* for the data in the electronic supplementary material, figure S2, is shown in (*b*). The linear regression for the six data points is shown by the line with a correlation coefficient value of approximate unity. A positive correlation coefficient value implies speed–accuracy trade-offs.

However, in a larger parameter space, different patterns were also observed. For each parameter set, we measured the Pearson correlation coefficient between *T* and *P* for the data points in the (*T*,*P*) space corresponding to different *H* values. For example, figure 4 contains the results for six *H* values. The relationship between *T* and *P* for the six *H* values is shown in figure 5*a*. The correlation coefficient calculated from the six data points is negative, which corresponds to the fact that the speed (i.e. 1/*T*) and accuracy (i.e. *P*) simultaneously improve or degrade as we vary *H*. By contrast, a positive correlation coefficient value, which can occur for other parameter values (figure 5*b*), implies speed–accuracy trade-offs. It should be noted that we employ a linear correlation coefficient to succinctly measure how *T* and *P* covary. The actual relationship between *T* and *P* is generally nonlinear, as shown in figure 5*a*. The methods for calculating the correlation coefficient are explained in more detail in appendix B.

**Figure 6.**
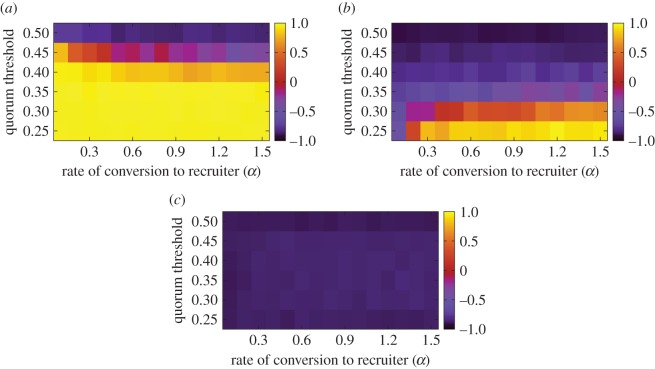
Correlation coefficient between the mean time to quorum, *T*, and the fraction of the correct choices, *P*, when we varied the fraction of high-threshold ants, *H*. (*a*) *α*_s_=0.01, (*b*) *α*_s_=0.1 and (*c*) *α*_s_=1. We set *N*=100, *α*_leak_=0.05 and *z*=0.3. The values of the quorum threshold shown are those normalized by the number of ants, *N*.

The correlation coefficient between *T* and *P* for various values of *α*, *α*_s_ and the quorum threshold is shown in figure 6. A small *α*_s_ value resulted in speed–accuracy trade-offs (i.e. a positive correlation coefficient) for the entire range of *α* and the quorum threshold that we explored (figure 6*a*). This pattern is opposite to that observed in figure 4. In fact, the accuracy (i.e. *P*) always increased with *H*. The speed decreased with *H* (i.e. *T* increased with *H*) when the value of *α*_s_ was small. This is consistent with intuition because high-threshold ants that happened to visit the poor nest site had to linger there for a long time before emigrating to the good nest site, and their votes were necessary to obtain a quorum in the good nest site. Even for the same value of *α*_s_ as that used in figure 4, speed–accuracy trade-offs took place if *α* was large or the quorum threshold was lowered (figure 6*b*). It should be noted that the parameter values used in figure 4, i.e. *α*=0.1 and a quorum threshold of 0.5, are located near the upper-left corner in figure 6*b*, where the speed and accuracy are positively correlated. When *α*_s_ was large, both the speed and accuracy increased with *H* (figure 6*c*).

**Figure 7.**
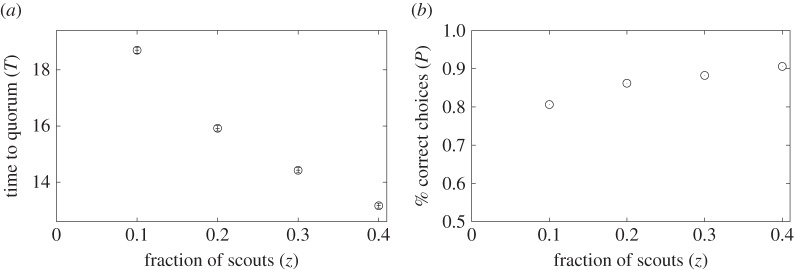
(*a*) Mean time to quorum, *T*, and (*b*) fraction of the correct choices, *P*, when we varied the fraction of scouts, *z*. We set *N*=100, *α*=0.1, *α*_s_=0.1, *α*_leak_=0.05 and *H*=0.2. The error bars in (*a*) represent the confidence intervals. In all cases, the error bars are smaller than the symbols showing the mean values.

**Figure 8.**
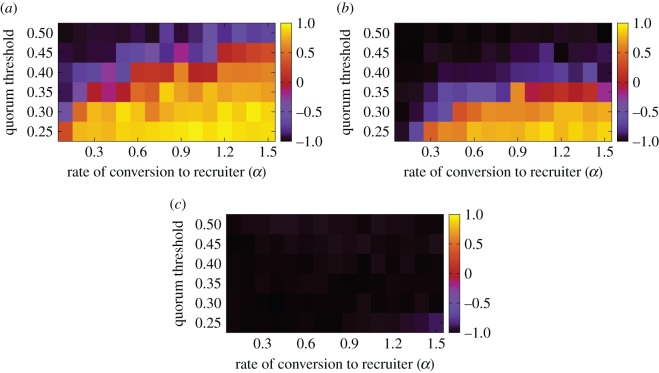
Correlation coefficient between the mean time to quorum, *T*, and the fraction of the correct choices, *P*, when we varied the fraction of scouts, *z*. (*a*) *α*_s_=0.01, (*b*) *α*_s_=0.1 and (*c*) *α*_s_=1. We set *N*=100, *α*_leak_=0.05 and *H*=0.2. The values of the quorum threshold shown are those normalized by *N*.

Ants may deploy more scouts initially under emergency than under normal conditions [35,28]. The dependence of *T* and *P* on the fraction of scouts, *z*, is shown in figure 7 with *α*=0.1, *α*_s_=0.1 and *H*=0.2. Both the speed and accuracy improved as *z* increased. This result is consistent with previous experimental results [39]. However, this pattern was again non-universal in the parameter space. The correlation coefficients for a range of *α*, *α*_s_ and the quorum threshold shown in figure 8 indicate the presence of speed–accuracy trade-offs (i.e. positive correlation coefficient in figure 8) when *α* is large, *α*_s_ is small and the quorum threshold is small, similar to when we varied *H* (figure 6).

In laboratory experiments, the quorum threshold is lower under emergency conditions than otherwise, probably enabling speed–accuracy trade-offs such that a high quorum threshold realizes slow but accurate decisions [9] (but this effect may be minor [35,7]). With *α*=0.1, *α*_s_=0.1, *H*=0.2 and *z*=0.3, the speed decreases (i.e. *T* increases) and the accuracy improves (i.e. *P* increases) as the quorum threshold increases (electronic supplementary material, figure S2). This result is consistent with the experimental findings [9]. The correlation between *T* and *P* was positive for the entire parameter region of *α*, *α*_s_ and *z* that we explored (electronic supplementary material, figure S3). This result indicates that speed–accuracy trade-offs when we vary the quorum threshold are robust against parameter variation.

We also examined the dependence of *T* and *P* on the rate at which high-threshold ants move from the poor to the good nest sites, *α*_s_. With *α*=0.1, *H*=0.2 and *z*=0.3, both the speed and accuracy improve as *α*_s_ increases (electronic supplementary material, figure S4). It should be noted that colonies could select the good nest site with a probability much larger than 0.5 (i.e. *P*≈0.7) even when high-threshold ants were not allowed to move from the poor to the good site (electronic supplementary material, figure S4*b*, at *α*_s_=0). The correlation between *T* and *P* is negative for the entire parameter region of *α*, quorum threshold, and the two values of *z* that we explored (electronic supplementary material, figure S5). This result indicates that the lack of speed–accuracy trade-offs is robust against parameter variation. These results contradict the experimental results in Robinson *et al.* [20], where setting *α*_s_=0 did not change either *T* or *P*, whereas *T* and *P* differ considerably between *α*_s_=0 and *α*_s_>0 in the model. The small number of colonies (6) used in this experiment made it difficult to tell if correct nest choice was signification at the colony level, despite colonies being able to choose the best nests. However, our model uses a much greater sample size and such effects may well only become manifest over a higher number of repeated trials.

### 3.3 Speed–cohesion trade-offs

In addition to speed–accuracy trade-offs, ant colonies show trade-offs between speed and cohesion in collective nest choice. When the new nest sites are of the same quality, colonies trade cohesion (i.e. individuals staying close together in one site) for speed when time urgency is high [30]. To examine if speed–cohesion trade-offs occur in our model, in this section we assume that all new nest sites are equally good. This is tantamount to assuming that all ants have the low threshold, i.e. *H*=0, and that all new nest sites are poor. In fact, these sites are all equally good such that all ants are satisfied with any site. In this situation, no ant is motivated to move from one nest site to another because ants that are once committed to a nest site do not switch to a different one in our model. Therefore, we set *α*_s_=0.

The cohesion is defined by

3.1 $$C=1-\frac{-\sum_{i=1}^{N_{\mathrm{nest}}}p_{i}\log p_{i}}{\log N_{\mathrm{nest}}},$$

where *N*_nest_ is the number of new nest sites, and p_*i*_ is the fraction of ants eventually staying in the *i*th nest site [30]. The entropy, $-\sum_{i=1}^{N_{\mathrm{nest}}}p_{i}\log p_{i}$, is large if ants are scattered in different nest sites with similar fractions. Equation (3.1) indicates that *C* is large if the entropy is small and vice versa. When p_*i*_=1/*N*_nest_ (1≤*i*≤*N*_nest_), the entropy is equal to $\log N_{\mathrm{nest}}$ such that the cohesion is the smallest, i.e. *C*=0. If all ants are in a single nest site, the entropy is equal to zero, and cohesion is maximized, i.e. *C*=1.

We ran simulations until 0.9*N* ants emigrated to a new nest site. It should be noted that we neglect the quorum rule and rapid transport after the quorum has been reached for simplicity. We denoted by *T*_f_ the time when 0.9*N* ants emigrated to a new nest for the first time. The values of *T*_f_ and *C* analysed in the following are the averages over the 10^4^ runs in which the threshold 0.9*N* was reached. We excluded the runs in which all the ants returned to the current nest due to leakage and terminated the dynamics. We set *α*_leak_=0.05. However, the following results were qualitatively the same when we turned off the leakage by setting *α*_leak_=0 (electronic supplementary material, figure S6).

**Figure 9.**
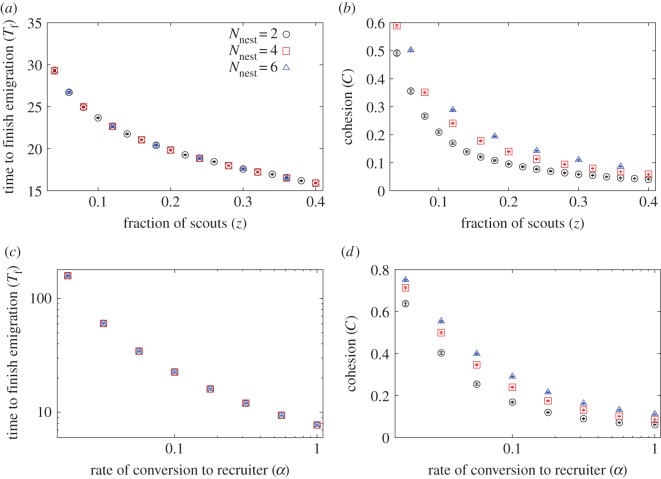
Speed–cohesion trade-offs. (*a*) Mean time to finish emigration, *T*_f_, and (*b*) cohesion, *C*, when we set *α*=0.1 and varied the fraction of scouts, *z*. (*c*) *T*_f_ and (*d*) *C* when we set *z*=0.12 and varied the rate at which committed ants turned into recruiters, *α*. In all cases, we set *N*=100, *α*_leak_=0.05, and the results for *N*_nest_=2, 4 and 6 are shown. The error bars represent the confidence intervals. In all cases, the error bars are smaller than the symbols showing the mean values.

Because homeless colonies deploy more scouts than those in intact nests [28,30,40,41,] (see [35] for similar results for a close species), we set *N*=100, *α*=0.1, varied the fraction of scouts, *z*, and measured the time to finish, *T*_f_, which is inversely proportional to the speed, and cohesion, *C*. Numerical results when *N*_nest_=2, 4 and 6 new nest sites have been presented are shown in figure 9. As we increased *z*, *T*_f_ and *C* both decreased, and the results did not depend on *N*_nest_ very much, consistent with speed–cohesion trade-offs. The results were qualitatively the same for *α*=0.02 (electronic supplementary material, figure S7*a*,*b*) and *α*=1 (electronic supplementary material, figure S7*c*,*d*).

Speed–cohesion trade-offs may occur as a result of different recruitment latencies between the two conditions. In other words, ants committed to a new nest site start recruiting earlier under high time urgency (i.e. home nest destroyed) than under low time urgency (i.e. home nest intact) [35,30]. To mimic this situation, we carried out another set of simulations in which we fixed *N*=100, *z*=0.12 and varied *α*. We chose this *z* value to make the number of scouts visiting each new site the same for each of *N*_nest_=2, 4 and 6. Ants start recruiting with a short latency when *α* is large, corresponding to an emergency situation, whereas a smaller *α* value emulates a normal situation. The dependence of *T*_f_ and *C* on *α* is shown in figure 9*c*,*d*, respectively. We found that when the time urgency was higher, the colony was less coherent, whereas the emigration occurred faster, consistent with speed–cohesion trade-offs. The results were qualitatively the same for *z*=0.36 (electronic supplementary material, figure S7*e*,*f*).

## 4. Discussion

Motivated by previous experimental studies [18,19], we developed a differential equation model and the corresponding agent-based model for collective nest choice when individual workers are assumed to have different acceptance thresholds in terms of the quality of nest sites. We observed speed–accuracy trade-offs as found in various laboratory experiments [9,28,29]. Trade-offs were robustly observed when the quorum threshold was varied in the numerical simulations (electronic supplementary material, figure S3), consistent with the experimental results [9,32] and predictions of a detailed agent-based model for bees [42]. Trade-offs were also observed when the fraction of high-threshold ants (figure 6) or the number of scouts (figure 8) was varied under the condition that high-threshold ants switched from the poor nest site to the good one relatively slowly or that the quorum threshold was low. In other parameter regions, the speed and accuracy simultaneously improved as a parameter varied, contrary to speed–accuracy trade-offs. However, the positive correlation between speed and accuracy as the number of scouts increased, as shown in our numerical simulations (figure 8), was in fact consistent with observations in laboratory experiments [39]. Last, when we assumed all new nest sites were of equal quality, we robustly observed speed–cohesion trade-offs consistent with those found in recent experiments [30].

There is also the potential to vary several of the other parameters used in our model in experimental scenarios. Recruitment latency may be altered, in effect, by changing the landmarks the ants have become familiar with so that recruiting ants can be made to take a more, or less, direct route back to the nest [43,44], thus increasing or decreasing the time taken to recruit nest-mates. Additionally, the proportion of scouting ants can be regulated by altering the quality of the current nest, as a higher proportion of the colony will engage in scouting behaviour when nest quality is lowered [41]. Another potential variable factor is the quorum threshold, as this can be artificially attained by adding ants to a prospective new nest [8]. Finally, in the laboratory, we have the ability to destroy the original nest or leave it intact [32], causing differences in the ability of colonies to fully assess multiple nest options [30]. By use of such techniques, we may be able to test the ability of the current model to correctly predict experimental outcomes, which will be valuable in further developing its efficacy as an investigative tool.

There are various differential equation and agent-based models for nest choice behaviour based on the quorum rule. Such previous models, except that in Robinson *et al.* [19], are different from the present model in that we examined the effect of heterogeneous acceptance thresholds across individuals, whereas previous models are concerned with different mechanisms. The previously examined mechanisms included the nest-dependent recruitment latency (i.e. roughly, the inverse of the acceptance rate or probability) [8,33–36,45–47], biased switching rates from one new nest site to another [8,33–36,45,46,] and lateral inhibition [48]. These models assume that ants that explore the arena before becoming quorate are homogeneous except that some individuals may be scouts and others are not. By contrast, we assumed that individual ants were inherently heterogeneous in their choosiness.

We previously proposed two models for the heterogeneous acceptance threshold hypothesis [19]. In the analytical, Markov chain variant of the model in Robinson *et al.* [19], ant individuals were modelled as different stochastic trajectories in the state space. In the analytical model, ants were implicitly assumed to be homogeneous such that, mathematically speaking, the collective nest choice in the model is driven by nest-dependent acceptance probabilities, not by ant-dependent acceptance thresholds. By contrast, the agent-based model [19] assumes that each ant is inherited with an acceptance threshold that is independently drawn from a common Gaussian distribution. The present models are complementary to the agent-based model [19] in that the former is parsimonious even without explicitly modelling the space, while the latter is better at quantitative fitting to experimental data. With both models, the colony could select a better nest site by the quorum rule. In addition, we showed with the present agent-based model that the heterogeneous acceptance thresholds can generate speed–accuracy and speed–cohesion trade-offs.

Many existing models of collective nest choice stand on the model proposed by Pratt *et al*. [8]. Based on their experimental data, they estimated the parameter values for the model [8]. In particular, they estimated the recruitment rate by tandem running per ant to be $0.033\;\min^{-1}$. The conversion rates from assessor to recruiter in the poor and the good sites per ant were set to 0.015 and $0.02\;\min^{-1}$, respectively. The switching rate from the poor to the good site per ant was set to $0.008\;\min^{-1}$. The switching rate from the good to the poor site per ant was set to zero. The assessors in their model and the committed ants in our model are not exactly the same (§2.1). In addition, we assumed mass interaction in the recruitment process, whereas Pratt *et al*. did not. Nevertheless, the parameter values derived by Pratt *et al*. serve as a guideline to narrow down plausible parameter values for our model. We normalized the time by assuming that the recruiting rate per recruiter–recruitee pair was equal to unity. Therefore, their parameter values yield the following rough estimates for our model: *α* around 0.015/0.033 and 0.02/0.033, i.e. *α*≈0.5 and *α*_s_≈0.008/0.033≈0.25. For these parameter values, our model predicts that speed–accuracy trade-offs do not occur when we vary the fraction of high-threshold ants, *H* (figure 6), or the fraction of scouts, *z* (figure 8).

In contrast to other modelling studies [7,8,34,35,45–47], we ignored the rapid transport phase after the quorum was reached, similarly to models in Marshall *et al.* [36]. With rapid transport, emigration proceeds three times faster than with tandem running, which is a slow recruitment process [8]. However, we do not consider that taking rapid transport into account in the model significantly affects the results. Both tandem running and rapid transport are mechanisms to amplify the difference between the number of votes for different options via positive feedback and can be modelled by a single nonlinear function in a unified manner [7,34,35,47]. Our claim is that this initial difference to be amplified can be created by the heterogeneous acceptance threshold. If the good site becomes quorate sooner than the poor site, the rapid transport that ensues would further magnify the difference in the number of ants selecting the two sites. Therefore, final nest choice is not expected to be changed by rapid transport.

By contrast, we assumed a recruiting process, contrasting to the agent-based model for the heterogeneous acceptance threshold hypothesis [19]. However, recruiting is not an essential component of our model. Without recruiting, the positive feedback will not operate. However, as long as some ants have a high threshold and switch from the current nest to the new nest site, the colony can select a good nest site with a high probability. We can easily model the lack of recruitment with the present model by setting *α*=0.

In both laboratory experiments [19,30] and in the field [38,1], ants may be subjected to more than two potential new nest sites. For simplicity, we did not consider this case except in §3.3, in which we examined speed–cohesion trade-offs. The main complication that arises when we examine this case in the present framework is that we will have to introduce multiple thresholds and hence more than two types of ants in general. For example, if three new sites of a good, intermediate and poor quality are presented, we need two thresholds and three types of ants. One threshold is located between good and intermediate quality, and the other is located between intermediate and poor quality. Highest-threshold ants are only satisfied with the good one. Intermediate-threshold ants are satisfied with either the good or intermediate one. Low-threshold ants (i.e. those effectively not using any threshold) are satisfied with any site. Even if the thresholds of individual ants are continuously distributed [19], it is sufficient to consider three types of ants if there are three nest sites of different quality (electronic supplementary material, figure S8), in much the same way as in the case of two nest sites (figure 1).

The importance of heterogeneous thresholds in colonies has been implicated with mathematical models. If colonies use heterogeneous acceptance thresholds to select nest sites, what else are they useful for? There are several lines of evidence that social insects employ heterogeneous thresholds when responding to external stimuli, to improve colony performance [49,50]. For example, in experiments with honeybees, it was suggested that heterogeneous response thresholds throughout the colony would promote a graded response to temperature fluctuations [21] (see [22,23] for bumblebees), consistent with the prediction of mathematical models for division of labour [49–52]. Heterogeneous acceptance thresholds in nest choice behaviour may also endow the colony with efficiency and robustness in a fluctuating environment. For example, with heterogeneous acceptance thresholds, if only poor nest sites are available and the colony is not subjected to an emergency situation, the colony may become quorate at one of those sites, but this would be a slow process, as high-threshold ants would remain unsatisfied and thus only low-threshold individuals would be able to contribute to the quorum. By contrast, in the presence of high-quality nest sites, as assumed in this study, a quorum for a high-quality site may be reached faster. If ants are homogeneous in their choosiness and use direct comparisons, such graded responses may be difficult. In addition, the threshold-based nest selection method requires fewer comparisons by individual ants, and therefore it is not as cognitively taxing as the direct comparison method. Furthermore, direct comparisons in general are more likely to lead to errors in judgement in various situations [53–55]. Thus, pursuing the functionality of heterogeneous acceptance thresholds with modelling and experimental approaches warrants further work.

## Supplementary Material

Supplementary figures for “Computational model of collective nest selection by ants with heterogeneous acceptance thresholds” by Masuda, O'Shea-Wheller, Doran, and Franks

## Appendix A. The good nest site is always chosen in the differential equation model

In this section, we show that the quorum for the good nest site is always reached before that for the poor nest site in our differential equation model with *α*_p_=*α*_g_(≡*α*). This result holds true irrespectively of the quorum threshold.

Let

A 1 $$\Delta x_{\mathrm{com}}\equiv x_{\ell ,g,\mathrm{com}}+x_{h,g,\mathrm{com}}-x_{\ell ,p,\mathrm{com}}$$

and

A 2 $$\Delta x_{\mathrm{rec}}\equiv x_{\ell ,g,\mathrm{rec}}+x_{h,g,\mathrm{rec}}-x_{\ell ,p,\mathrm{rec}}.$$

Our first goal is to show Δ*x*_com_,Δ*x*_rec_>0 for all *t*>0.

By combining equations (2.3), (2.5) and (2.6), we obtain

A 3 $$\frac{d\Delta x_{\mathrm{com}}}{dt}=(x_{\ell ,g,\mathrm{rec}}+x_{h,g,\mathrm{rec}})(x_{\ell ,c}+x_{g,c})+\alpha_{s}x_{h,p,\mathrm{vis}}-x_{\ell ,p,\mathrm{rec}}x_{\ell ,c}-(\alpha +\alpha_{\mathrm{leak}})\Delta x_{\mathrm{com}}.$$

Equation (A 3) with the initial condition Δ*x*_com_=*Hz*/2 for *t*=0 leads to

A 4 $$\begin{array}{rl} \Delta x_{\mathrm{com}} & =\frac{Hz}{2}e^{-(\alpha +\alpha_{\mathrm{leak}})t}+\int_{0}^{t}e^{\left(\alpha +\alpha_{\mathrm{leak}})(t^{\prime }-t\right)}\{[x_{\ell ,g,\mathrm{rec}}(t^{\prime })+x_{h,g,\mathrm{rec}}(t^{\prime })][x_{\ell ,c}(t^{\prime })+x_{h,c}(t^{\prime })]\\ & \;+\alpha_{s}x_{h,p,\mathrm{vis}}(t^{\prime })-x_{\ell ,p,\mathrm{rec}}(t^{\prime })x_{\ell ,c}(t^{\prime })\}\;dt^{\prime }\\ & >\frac{Hz}{2}e^{-(\alpha +\alpha_{\mathrm{leak}})t}+\int_{0}^{t}e^{\left(\alpha +\alpha_{\mathrm{leak}})(t^{\prime }-t\right)}\Delta x_{\mathrm{rec}}(t^{\prime })[x_{\ell ,c}(t^{\prime })+x_{h,c}(t^{\prime })]\;dt^{\prime }, \end{array}$$

where we explicitly represented by the argument *t*^′^ the fact that the variables in the integral were evaluated at time *t*^′^. We used *x*_h,p,vis_>0 and −*x*_ℓ,p,rec_*x*_ℓ,c_>−*x*_ℓ,p,rec_(*x*_ℓ,c_+*x*_h,c_) for *t*>0 to derive the last inequality in equation (A 4).

Using equations (2.7)–(2.9), we obtain

A 5 $$\frac{d\Delta x_{\mathrm{rec}}}{dt}=\alpha \Delta x_{\mathrm{com}}-\alpha_{\mathrm{leak}}\Delta x_{\mathrm{rec}}.$$

Because Δ*x*_rec_=0 when *t*=0, combination of equations (A 4) and (A 5) yields

A 6 $$\begin{array}{rl} \Delta x_{\mathrm{rec}} & =\alpha \int_{0}^{t}e^{\alpha_{\mathrm{leak}}(t^{\prime }-t)}\Delta x_{\mathrm{com}}(t^{\prime })\;dt^{\prime }\\ & >\alpha \int_{0}^{t}e^{\alpha_{\mathrm{leak}}(t^{\prime }-t)}\left[\frac{Hz}{2}e^{-(\alpha +\alpha_{\mathrm{leak}})t^{\prime }}+\int_{0}^{t^{\prime }}e^{\left(\alpha +\alpha_{\mathrm{leak}})(t^{\prime \prime }-t^{\prime }\right)}\Delta x_{\mathrm{rec}}(t^{\prime \prime })[x_{\ell ,c}(t^{\prime \prime })+x_{h,c}(t^{\prime \prime })]\;dt^{\prime \prime }\right]\;dt^{\prime }. \end{array}$$

Equation (A 6) implies Δ*x*_rec_>0 (*t*>0). Combination of Δ*x*_rec_>0 (*t*>0) and equation (A 4) implies Δ*x*_com_>0 (*t*>0).

We define

A 7 $$\begin{array}{rl} \Delta x_{\mathrm{vote}} & \equiv (x_{\ell ,g,\mathrm{com}}+x_{h,g,\mathrm{com}}+x_{\ell ,g,\mathrm{rec}}+x_{h,g,\mathrm{rec}})-(x_{\ell ,p,\mathrm{com}}+x_{\ell ,p,\mathrm{rec}}+x_{h,p,\mathrm{vis}})\\ & =\Delta x_{\mathrm{com}}+\Delta x_{\mathrm{rec}}-x_{h,p,\mathrm{vis}}, \end{array}$$

which is the difference between the vote for the good nest site and that for the poor one. By combining equations (2.4), (A 3) and (A 5), we obtain

A 8 $$\frac{d\Delta x_{\mathrm{vote}}}{dt}=\Delta x_{\mathrm{rec}}(x_{\ell ,c}+x_{h,c})+2\alpha_{s}x_{h,p,\mathrm{vis}}-\alpha_{\mathrm{leak}}\Delta x_{\mathrm{vote}}.$$

Because Δ*x*_vote_=0 when *t*=0, we obtain from equation (A 8)

A 9 $$\begin{array}{rl} \Delta x_{\mathrm{vote}} & =\int_{0}^{t}e^{-\alpha_{\mathrm{leak}}(t^{\prime }-t)}\left\{\Delta x_{\mathrm{rec}}(t^{\prime })\left[x_{\ell ,c}(t^{\prime })+x_{h,c}(t^{\prime })\right]+2\alpha_{s}x_{h,p,\mathrm{vis}}\right\}\;dt^{\prime }>0 \end{array}$$

when *t*>0. In other words, the vote for the good site is larger than that for the poor site any time. Therefore, the quorum threshold is reached for the good site prior to the poor site for an arbitrary quorum threshold.

## Appendix B. Definition of the correlation coefficient

We measured the correlation between *T* and *P* shown in figures 6 and 8, and the electronic supplementary material, figures S1, S3 and S5, as follows. In figure 6, we fixed *α*, *α*_s_ and the quorum threshold, and ran the simulation 10^4^ times for each of *H*=0, 0.0667, 0.1333, 0.2, 0.2667, 0.3333 and 0.4. These *H* values correspond to the number of scouts to the good nest site equal to 1, 2, 3, 4, 5 and 6, respectively. For each *H*, we obtained *T* and *P* as the averages over the 10^4^ runs. Then, we regarded the (*T*, *P*) pairs as six data points and calculated the Pearson correlation coefficient. In figure 8, we calculated the correlation coefficient for given *α*, *α*_s_ and the quorum threshold on the basis of four data points corresponding to *z*=0.1, 0.2, 0.3 and 0.4. With the largest value of *z* (i.e. *z*=0.4), 0.2*N*(=20) scouts initially visited each new nest site. It should be noted that this number is smaller than the smallest quorum threshold examined in the figure (i.e. 0.25). This treatment was to ensure that the quorum was not reached in the initial condition. In the electronic supplementary material, figures S3 and S5, we calculated the correlation coefficient on the basis of the six data points corresponding to the quorum threshold values of 0.25, 0.3, 0.35, 0.4, 0.45 and 0.5, and the six data points corresponding to *α*_s_=0, 0.1, 0.2, 0.3, 0.4 and 0.5, respectively.
