# Supplementary material for: Computational model of collective nest selection by ants with heterogeneous acceptance thresholds
Source: R Soc Open Sci. 2015 Jun 9;2(6):140533. doi: 10.1098/rsos.140533 (PMC4632542; doi:10.1098/rsos.140533)
Supplement: Supplementary figures for “Computational model of collective nest selection by ants with heterogeneous acceptance thresholds” by Masuda, O'Shea-Wheller, Doran, and Franks [file rsos140533supp1.pdf]

Supplementary figures for

"Computational model of collective nest selection by ants with heterogeneous acceptance thresholds"

by Masuda, O'Shea-Wheller, Doran, and Franks

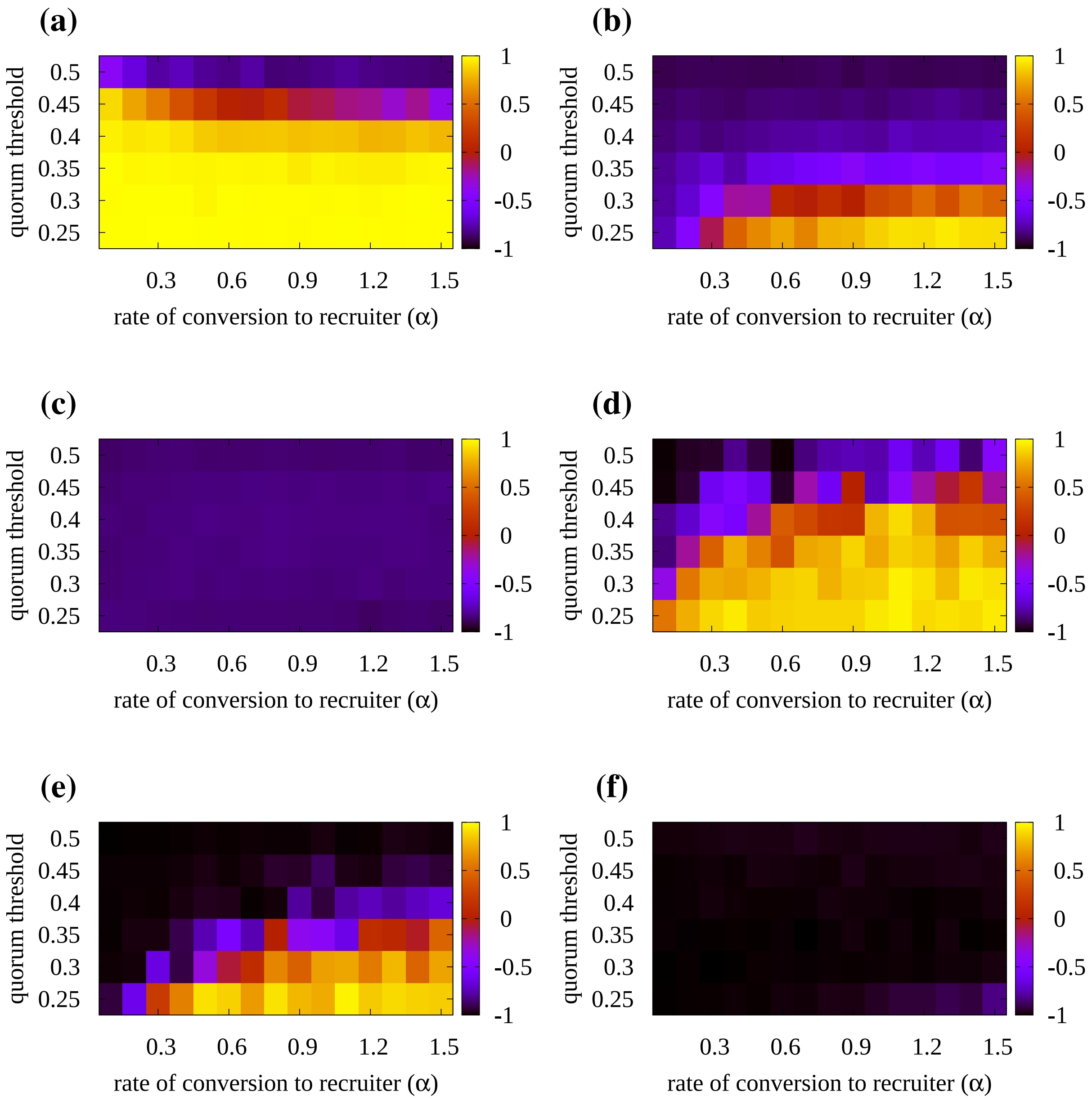
(a)
(b)
(c)
(d)
(e)
(f)
quorum threshold
0.5
0.45
0.4
0.35
0.3
0.25
0.3
0.6
0.9
1.2
1.5
rate of conversion to recruiter (α)
1
0.5
0
-0.5
-1

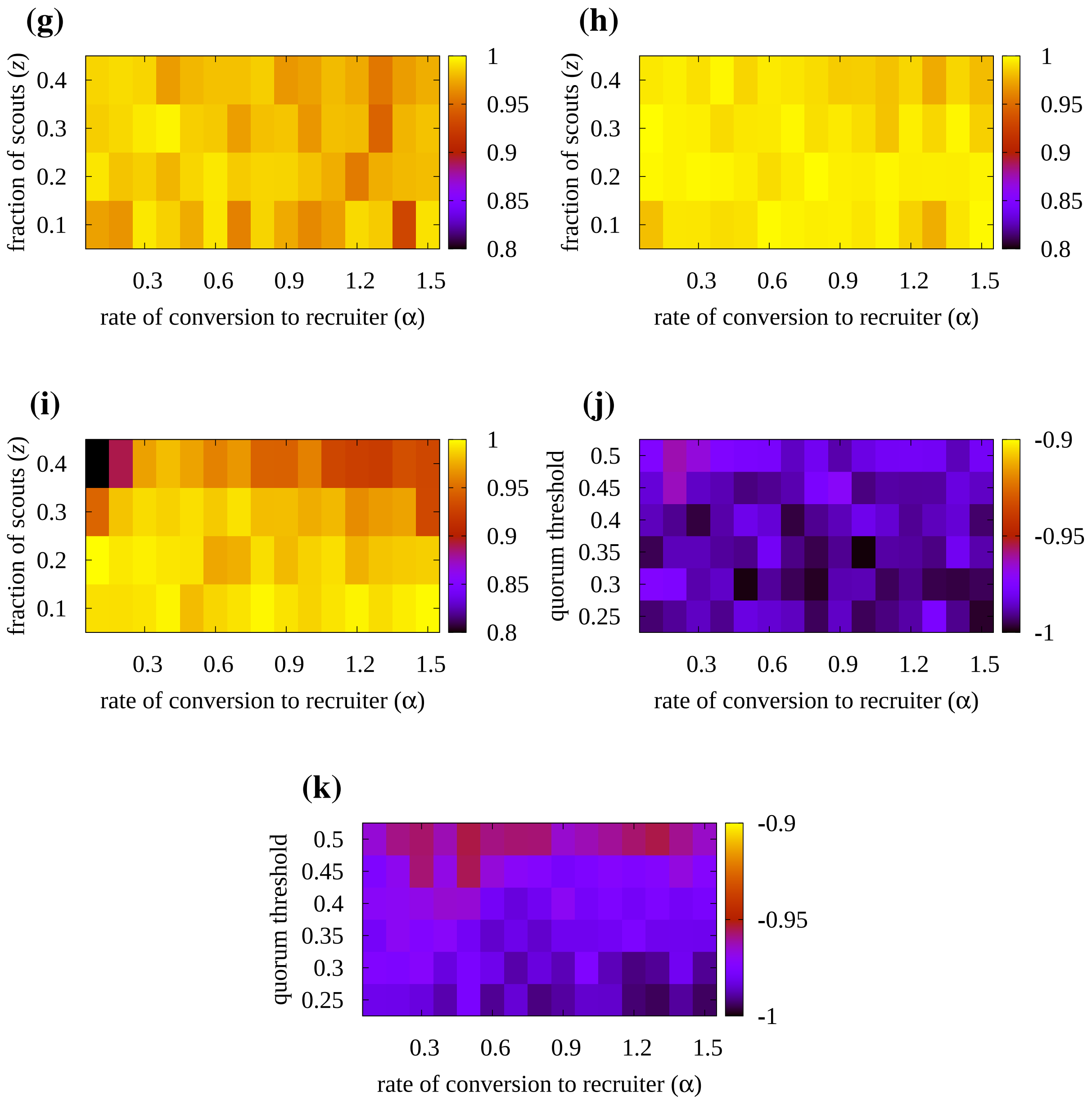

Supplementary Fig. S1: Speed-accuracy trade-offs when leakage is absent. All panels show the correlation coefficient between the mean time to quorum, T, and the fraction of the correct choices, P, when $N = 100$ and $\alpha_{\mathrm{leak}} = 0$. In (a), (b), and (c), we set $z = 0.3$ and varied the fraction of high-threshold ants, H, for each pair of α and the quorum threshold. (a) $\alpha_{\mathrm{s}} = 0.01$, (b) $\alpha_{\mathrm{s}} = 0.1$, and (c) $\alpha_{\mathrm{s}} = 1$. In (d), (e), and (f), we set $H = 0.2$ and varied the fraction of scouts, z, for each pair of α and the quorum threshold. (d) $\alpha_{\mathrm{s}} = 0.01$, (e) $\alpha_{\mathrm{s}} = 0.1$, and (f) $\alpha_{\mathrm{s}} = 1$. In (g), (h), and (i), we set $H = 0.2$ and varied the quorum threshold for each pair of α and z. (g) $\alpha_{\mathrm{s}} = 0.01$, (h) $\alpha_{\mathrm{s}} = 0.1$, and (i) $\alpha_{\mathrm{s}} = 1$. In (j) and (k), we set $H = 0.2$ and varied the rate of switching to the good nest, α_{s}, for each value of α and the quorum threshold. (j) $z = 0.1$ and (k) $z = 0.3$. The values of the quorum threshold shown are those normalised by N.

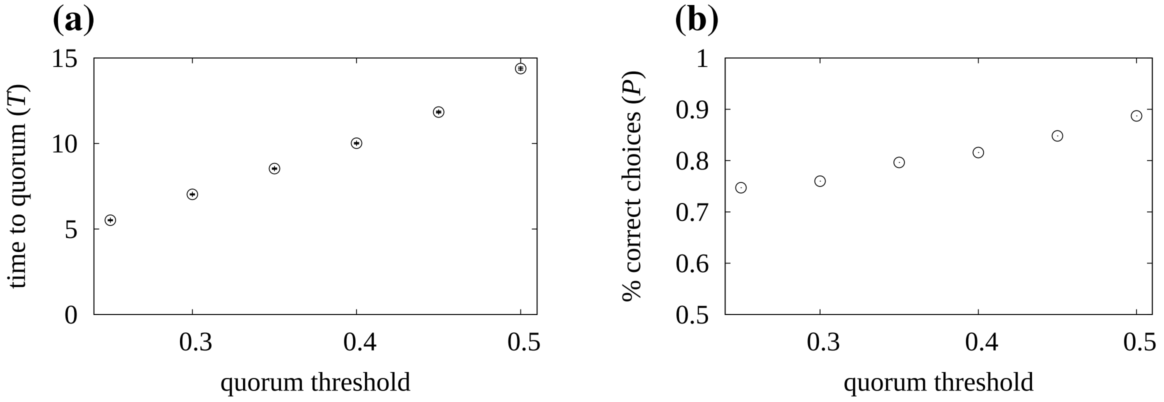

Supplementary Fig. S2: (a) Mean time to quorum, T, and (b) fraction of the correct choices, P, when we varied the quorum threshold. We set $N = 100$, $\alpha = 0.1$, $\alpha_{\rm s} = 0.1$, $\alpha_{\rm leak} = 0.05$, $H = 0.2$, and $z = 0.3$. The error bars in (a) represent the confidence intervals. In all cases, the error bars are smaller than the symbols showing the mean values.

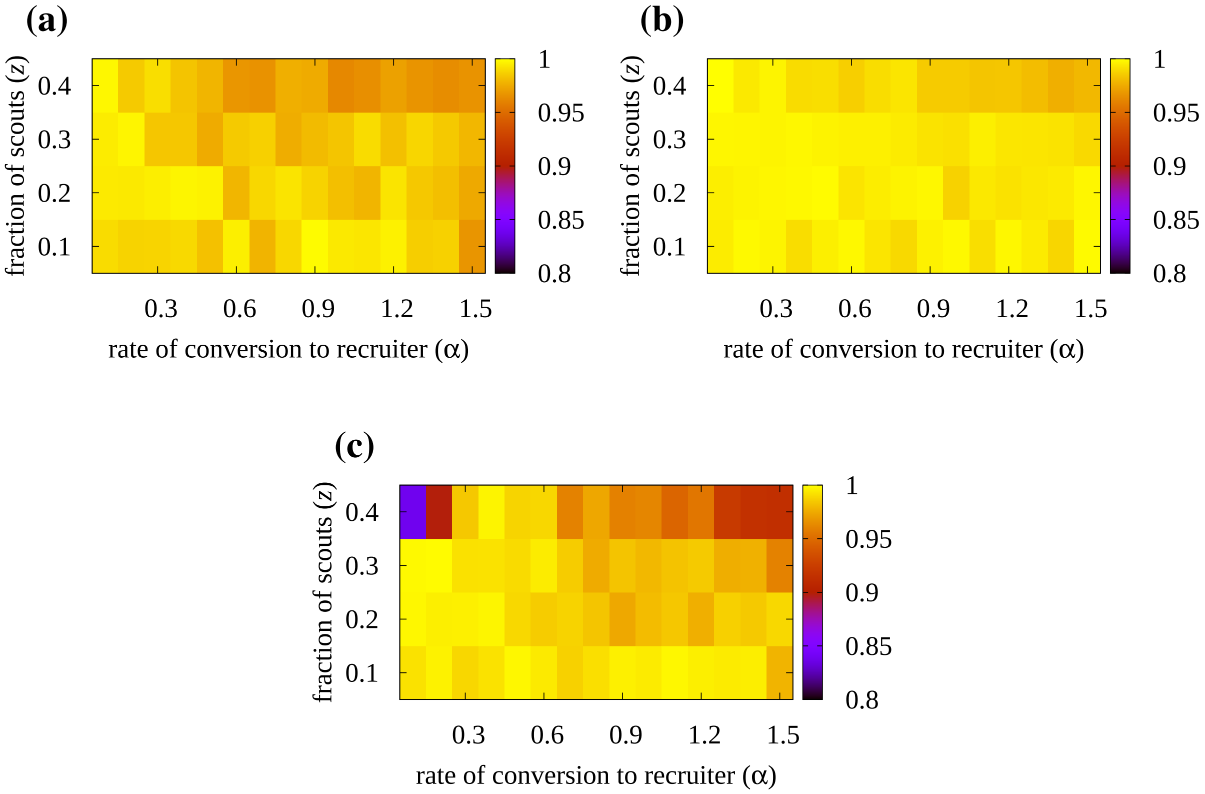

Supplementary Fig. S3: Correlation coefficient between the mean time to quorum, T, and the fraction of the correct choices, P, when we varied the quorum threshold. (a) $\alpha_{\rm s} = 0.01$, (b) $\alpha_{\rm s} = 0.1$, and (c) $\alpha_{\rm s} = 1$. We set $N = 100$, $\alpha_{\rm leak} = 0.05$, and $H = 0.2$. The values of the quorum threshold shown are those normalised by N.

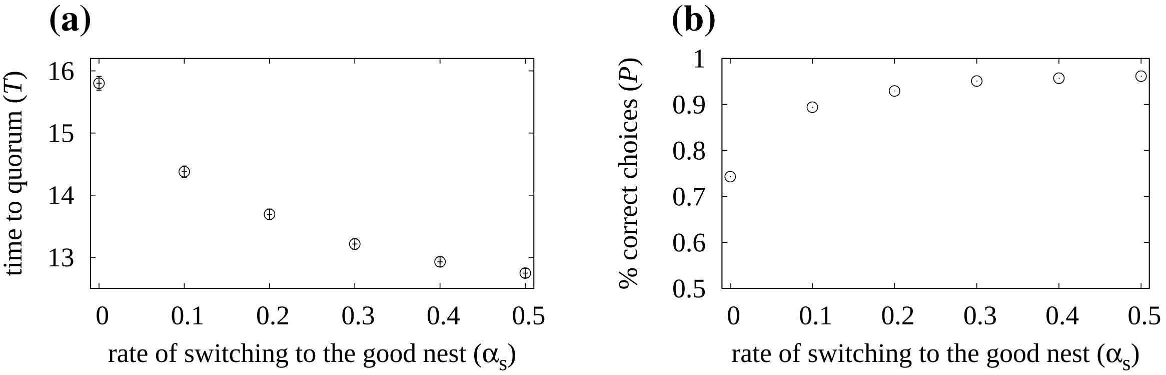

Supplementary Fig. S4: (a) Mean time to quorum, T, and (b) fraction of the correct choices, P, when we varied the rate of switching to the good nest, α_{s}. We set $N = 100$, $\alpha = 0.1$, $\alpha_{\mathrm{leak}} = 0.05$, $H = 0.2$, and $z = 0.3$. The error bars in (a) represent the confidence intervals.

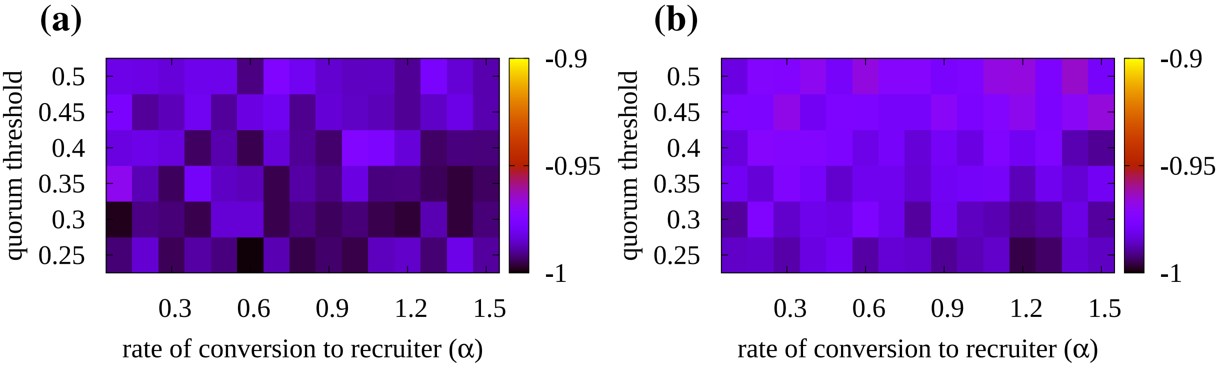

Supplementary Fig. S5: Correlation coefficient between the mean time to quorum, T, and the fraction of the correct choices, P, when we varied the rate of switching to the good nest, α_s. (a) $z = 0.1$ and (b) $z = 0.3$. We set $N = 100$, $\alpha_\mathrm{leak} = 0.05$, and $H = 0.2$. The values of the quorum threshold shown are those normalised by N.

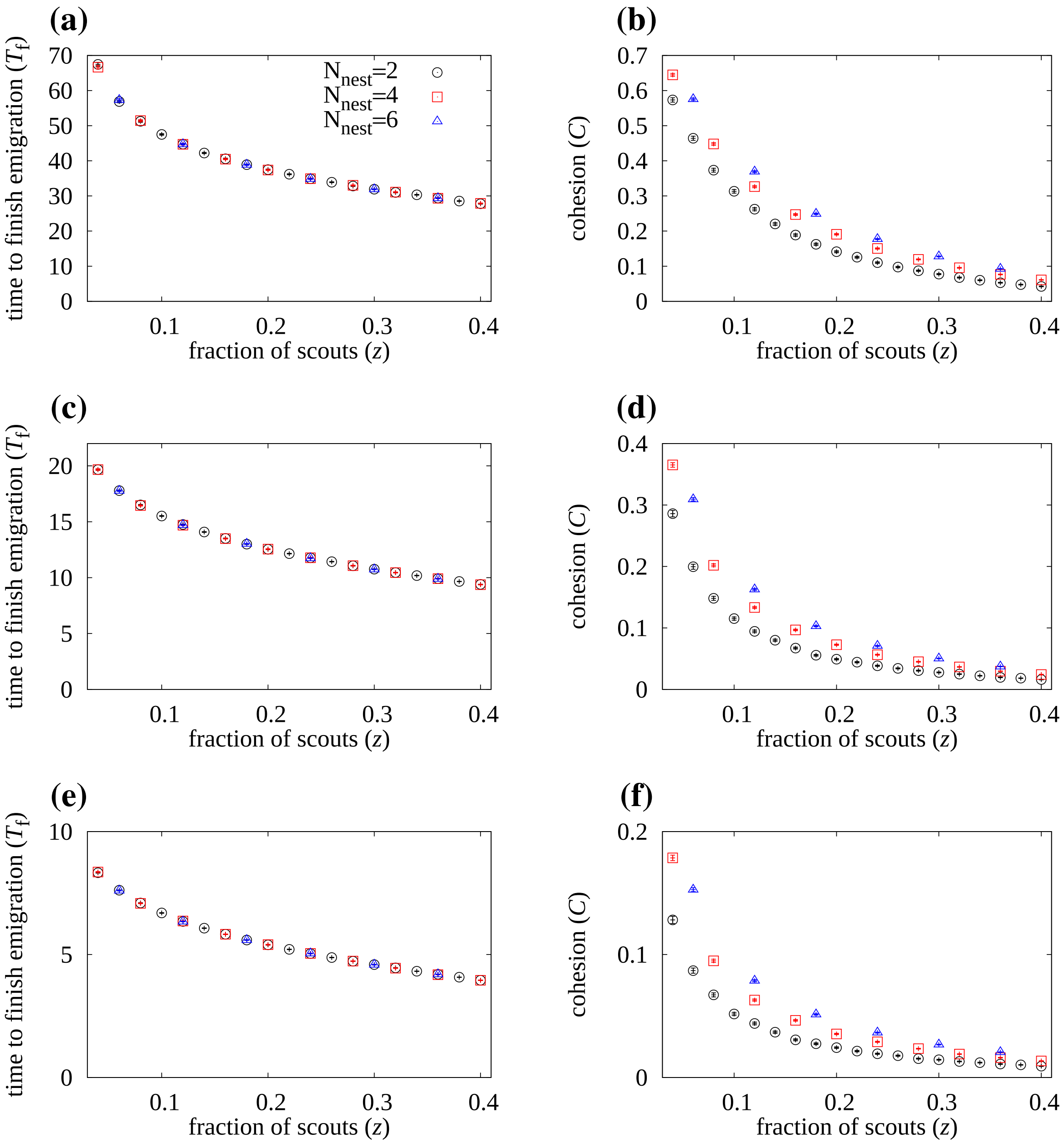

(a)
(b)
(c)
(d)
(e)
(f)
time to finish emigration (T_f)
cohesion (C)
fraction of scouts (z)
N_{nest}=2
N_{nest}=4
N_{nest}=6

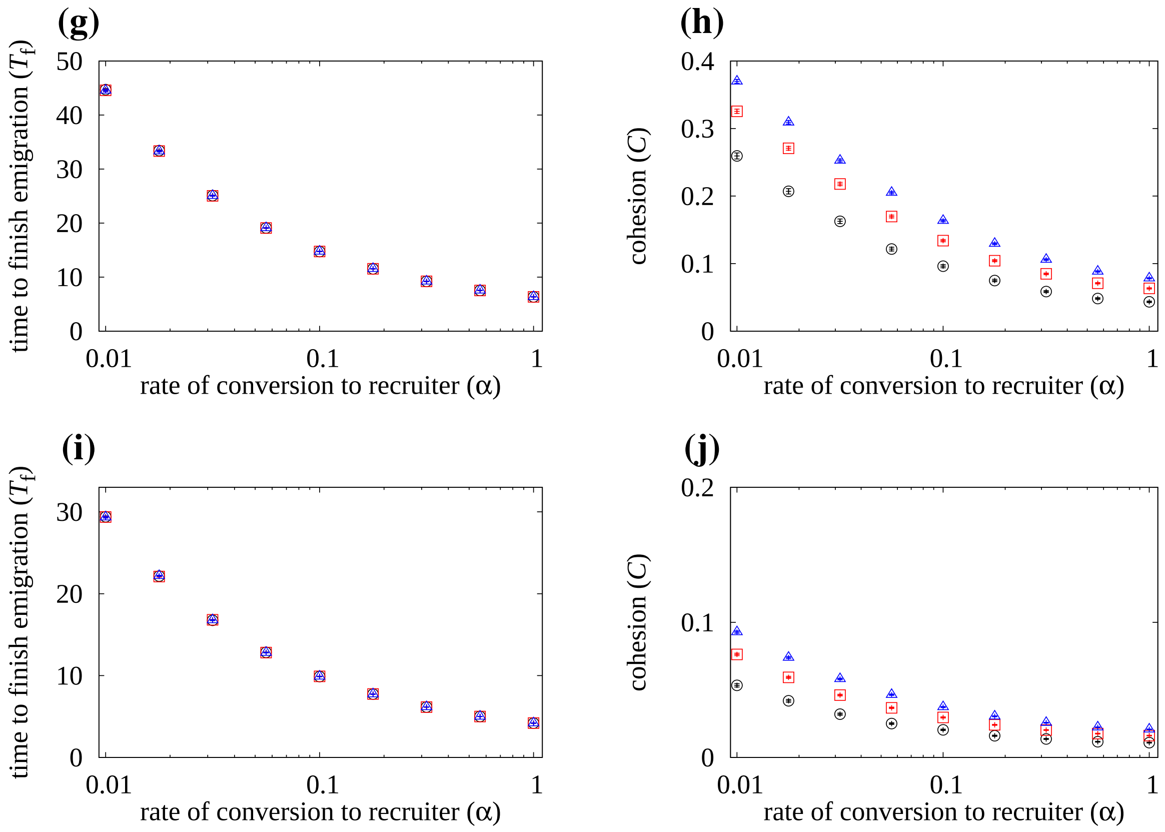

Supplementary Fig. S6: Speed-cohesion trade-offs when leakage is absent. We ran each simulation until the current nest became empty, i.e., until each ant arrived at a new nest site. This event always occurred because of the absence of leakage. (a) Mean time to finish emigration, T_{f}, and (b) cohesion, C, when we set $\alpha = 0.01$ and varied the fraction of scouts, z. (c) T_{f} and (d) C when we set $\alpha = 0.1$ and varied z. (e) T_{f} and (f) C when we set $\alpha = 1$ and varied z. (g) T_{f} and (h) C when set set $z = 0.12$ and varied the rate at which the committed ant turns into recruiter, α. (i) T_{f} and (j) C when we set $z = 0.36$ and varied α. In all panels we set $N = 100$, $\alpha_{\mathrm{leak}} = 0$, and $N_{\mathrm{nest}} = 2$, 4, and 6. The error bars represent the confidence intervals. In all cases, the error bars are smaller than the symbols showing the mean values. It should be noted that the minimum value of α when we varied z is set to 0.01 ((a) and (b)), not 0.02 as was the case in the presence of leakage (Fig. S7(a) and (b)). This is because no run has reached the quorum with $\alpha = 0.01$ in the presence of leakage (i.e., $\alpha_{\mathrm{leak}} = 0.05$).

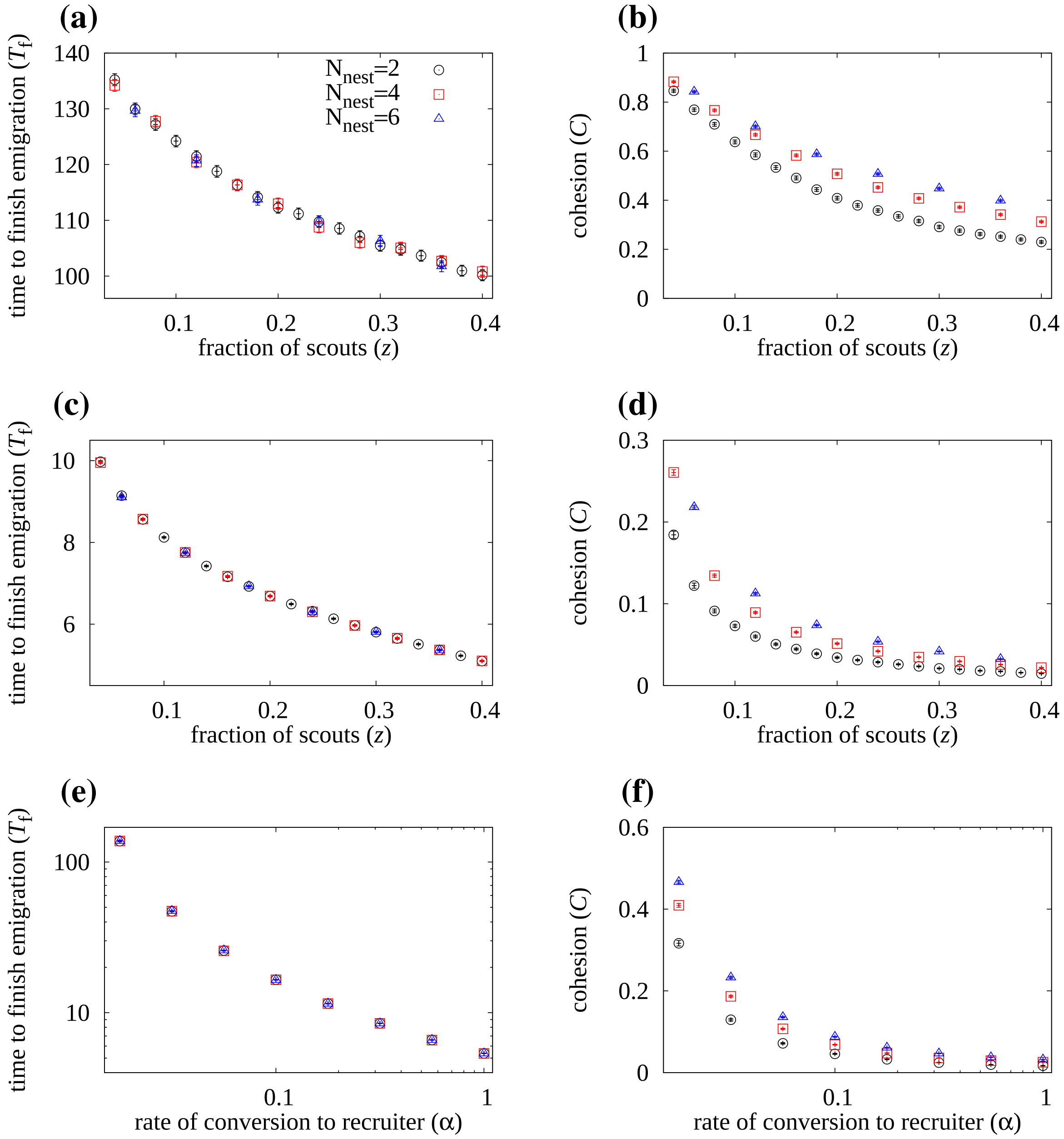

Supplementary Fig. S7: Speed-cohesion trade-offs for different parameter values. (a) Mean time to finish emigration, T_{f}, and (b) cohesion, C, when we set $\alpha = 0.02$ and varied the fraction of scouts, z. (c) T_{f} and (d) C when we set $\alpha = 1$ and varied z. (e) T_{f} and (f) C when we set $z = 0.36$ and varied the rate at which the committed ant turns into recruiter, α. In all panels, we set $N = 100$, $\alpha_{\mathrm{leak}} = 0.05$, and $N_{\mathrm{nest}} = 2$, 4, and 6. The error bars represent the confidence intervals. Except in some cases in (a), the error bars are smaller than the symbols showing the mean values.

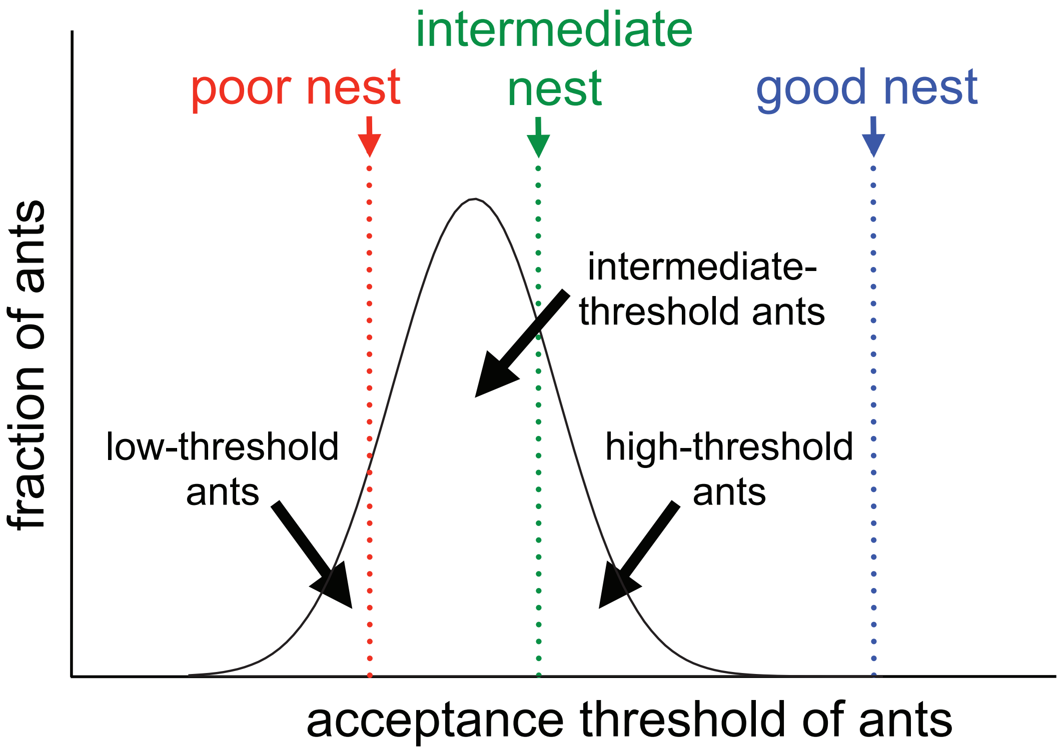

Supplementary Fig. S8: The normal distribution of the threshold can be mapped to a three-valued threshold distribution when there are three nest sites. Ants having a threshold larger than the quality of the intermediate nest are high-threshold ants. Ants having a threshold between the quality of the poor nest and that of the intermediate nest are intermediate-threshold ants. Ants having a threshold smaller than the quality of the poor nest are low-threshold ants.
